# Dr. Addison's New Post

**Published:** 1914-08-15

**Authors:** 


### Dr. Addison's New Post.

It was officially announced on Tuesday last that Dr. Christopher Addison, M.P., had been appointed Parliamentary Secretary to the Board of Education. At any other time than that of the present crisis this political appointment would rouse considerable comment among members of the medical profession both on and off the panel. Still, however, medical men, however busy, will not fail to note how Dr. Addison has proved to be the favoured of the politicians ever since the negotiations between the British Medical Association and the Chancellor of the Exchequer over the profession's attitude towards the Insurance Act ended in the doctors breaking their ranks. Probably Dr. Addison himself would be the last to deny that his new post is the latest reward for political services rendered, of which acute observers were ready to prophesy no long delay after the Chancellor's official benediction to him was given at the dinner in his honour some months ago.

